# The Benefits of Closed-Loop Transcranial Alternating Current Stimulation on Subjective Sleep Quality

**DOI:** 10.3390/brainsci8120204

**Published:** 2018-11-22

**Authors:** Charles S. H. Robinson, Natalie B. Bryant, Joshua W. Maxwell, Aaron P. Jones, Bradley Robert, Melanie Lamphere, Angela Combs, Hussein M. Al Azzawi, Benjamin C. Gibson, Joseph L. Sanguinetti, Nicholas A. Ketz, Praveen K. Pilly, Vincent P. Clark

**Affiliations:** 1 Psychology Clinical Neuroscience Center, Department of Psychology, Logan Hall, MSC03-2220, 1 University of New Mexico, Albuquerque, NM 87131-0001, USA; csrobinson@mrn.org (C.S.H.R.); nbbryant@gmail.com (N.B.B.); maxwellj@unm.edu (J.W.M.); aaronjones@unm.edu (A.P.J.); brobert@unm.edu (B.R.); zia100yrs@unm.edu (M.L.); angelacombs11@gmail.com (A.C.); azzawi@unm.edu (H.M.A.A.); bencookgibson@gmail.com (B.C.G.); jay.sanguinetti@gmail.com (J.L.S.); 2 The Mind Research Network and LBERI, 1101 Yale Blvd. NE, Albuquerque, NM 87106, USA; 3 Center for Human-Machine Collaboration, Information and Systems Sciences Laboratory, HRL Laboratories, 3011 Malibu Canyon Road, Malibu, CA 90265, USA; nick.ketz@gmail.com

**Keywords:** Karolinska Sleep Diary, tACS, closed-loop, slow-wave sleep, sleep efficiency, electroencephalogram

## Abstract

Background: Poor sleep quality is a common complaint, affecting over one third of people in the United States. While sleep quality is thought to be related to slow-wave sleep (SWS), there has been little investigation to address whether modulating slow-wave oscillations (SWOs) that characterize SWS could impact sleep quality. Here we examined whether closed-loop transcranial alternating current stimulation (CL-tACS) applied during sleep impacts sleep quality and efficiency. Methods: CL-tACS was used in 21 participants delivered at the same frequency and in phase with endogenous SWOs during sleep. Sleep quality was assessed in the morning following either verum or sham control stimulation during sleep, with order counterbalanced within participants. Results: Higher sleep quality and efficiency were found after verum stimulation nights compared to control. The largest effects on sleep quality were found immediately following an adaptation night in the laboratory for which sleep quality was reduced. Conclusions: Applying CL-tACS at the same frequency and phase as endogenous SWOs may offer a novel method to improve subjective sleep quality after a night with poor quality sleep. CL-tACS might be helpful for increasing sleep quality and efficiency in otherwise healthy people, and in patients with clinical disorders that involve sleep deficits.

## 1. Introduction

According to a 2014 report by the Centers for Disease Control and Prevention (CDC), 35.2% of adults in the United States get fewer than 7 h of sleep per night [1]. The National Sleep Foundation’s 2014 Sleep in America poll [2] states that, of those who are getting sufficient sleep, 35% rate their sleep quality “only fair” or “poor.” Further, 20% of Americans report not waking up feeling refreshed on any day in the past week, and 67% of people who reported poorer sleep quality are also likely to report poor health. Poor sleep quality is also related to stress and life satisfaction, productivity loss, absenteeism, and work-related injuries [3]. Sleep-related injuries are estimated at 110,000 per year, 5000 of which are accidents involving commercial vehicles [4]. 

Research related to improving poor sleep quality has the potential for a large impact on these and other disorders. Current treatments depend in part on detailed analysis of conditions for each patient, and involve pharmaceutical and/or behavioral therapies, including supplements such as tryptophan and melatonin [5], behavioral techniques such as cognitive behavioral therapy [6], devices such as continuous positive airway pressure [7] and cranial nerve stimulation [6,8] and light-based interventions [9], among others. While all of these have demonstrated some benefits for certain patient populations, none have been found to produce large and reliable benefits on sleep quality in the majority of people.

Research suggests subjective sleep quality is positively correlated with time spent in stage 3 of non-rapid eye movement (NREM) sleep, also referred to as slow-wave sleep (SWS) [10]. Prior studies suggest that non-invasive brain stimulation (NIBS) may be used to benefit various aspects of sleep. The first of these was the finding that transcranial electrical stimulation (tES) during SWS increased time spent in SWS [11,12]. Other research applying bi-frontal transcranial direct current stimulation (tDCS) to modulate Total Sleep Time (TST) and sleep efficiency, found that these were reduced after verum tDCS relative to control [13]. Another study [14] found that bilateral open-loop transcranial alternating current stimulation tACS over fronto-temporal areas did not cause changes in daytime sleepiness. Brain stimulation has also been found to modulate ongoing sleep electroencephalogram (EEG) activity [15,16], which may interact with sleep efficiency and subjective sleep quality. However, this has not been examined in previous studies, and no positive effects have yet been reported. In the present study, the effects of closed-loop transcranial alternating current stimulation (CL-tACS) designed to match the frequency and phase of endogenous slow-wave oscillations SWOs during sleep were investigated on sleep efficiency and subjective sleep quality. It was found that sleep quality and efficiency were enhanced during CL-tACS stimulation relative to control nights.

## 2. Materials and Methods

### 2.1. Participants 

This data was collected as part of a larger project using transcranial electrical stimulation (tES) to enhance memory consolidation [17]. Inclusion criteria were 18–40 years of age, native English speaker, right handed and regularly goes to bed before midnight. Exclusion criteria included (1) Using any form of nicotine excessively; (2) History of head injury with loss of consciousness for more than 5 min; (3) Uncorrected hearing or vision impairment, including color blindness; (4) Intelligence quotient (IQ) scores on the Shipley-2 IQ test of less than 90; (5) Current or history of significant neurological or psychiatric illness, including drug dependence; (6) Medication or drug use judged likely to change brain activity, behavior or sleep cycle; (7) Hair style that reduces quality of EEG and/or stimulation; (8) Typically active at night (work, studying, etc.); (9) Diagnosed sleep disorder or excessive difficulty falling or staying asleep; (10) Normally takes prescription or over the counter sleep aids to go to sleep; (11) Usually takes daytime naps; (12) Consumed alcohol or caffeine during afternoon before experimental nights; (13) Unusual sleep patterns observed during first adaptation night in the laboratory, including less than 30 min of stage 3 sleep or less than 40 min of stage 2 and 3 sleep in the first sleep cycle.

Participants were given the Karolinska Sleep Diary (KSD) [18] to record their pre-laboratory sleep quality at home. Next, the first adaptation night was performed for subjects to acclimate to sleeping in the laboratory, then slept in the laboratory the subsequent night and a third night separated by an average of 5.24 days (range 3 to 11) from the second night. The stimulation condition was counterbalanced between the two experimental nights (namely, nights 2 and 3). Group “V/C” (Verum/Control) received stimulation on Night 2 (the second night, immediately following adaptation night), and Group “C/V” (Control/Verum) received stimulation on Night 3 (with no re-adaptation). Five of the original 26 healthy participants were excluded from the analyses (1 from the V/C group and 4 from the C/V group) due to technical difficulties in the delivery of brain stimulation during sleep. The remaining 21 participants were included in the present analyses (mean age of 20.14, 7 female). All participants were given monetary compensation and provided signed informed consent to participate in the study as approved by the Chesapeake Institutional Review Board (protocol 2015-837529).

### 2.2. Design and Procedure

Participants rated their sleep quality using the Karolinska Sleep Diary, a 12-item questionnaire assessing specific aspects of sleep (e.g., bed and wake times, number of awakenings) and global indicators of sleep quality (e.g., ease of falling asleep, feeling refreshed) [19]. Sleep quality was assessed in the mornings following pre-adaptation, adaptation, and the two nights of tACS intervention at either verum (1.5 mA per stimulating electrode) or control (0 mA). Five participants received verum stimulation at a lower dose (0.52 mA per electrode). Sensitivity analyses were performed with and without these participants as explained below.

As noted above, the first night of sleep in the laboratory was an adaptation night (night 1) followed by the second night of sleep in the laboratory, which was when the participants experienced either verum or control stimulation. The third night of sleep in the laboratory, the remaining tACS condition was administered. The assignment to the intervention was randomized across subjects and counterbalanced, and participants were blind to the order of the stimulation condition in their assignments. This is corroborated by the findings of no significant differences comparing the verum and control nights of sleep for each group and comparing the number of awakenings for each group at Night 2 and Night 3.

### 2.3. Stimulation Protocol

Verum or control tDCS was applied during a learning task administered during the afternoon before nights 2 and 3 (described in [17]). Anodal tDCS was applied to location F10, with the cathode placed on the left arm. Stimulation was 2.0 mA applied for 30 min during training. The same condition of verum or control tDCS was applied during the afternoon as verum or control CL-tACS during the subsequent night.

EEG recording and stimulation were administered using a Neuroelectrics StarStim32 system. Electrodes were affixed to the scalp using a neoprene cap following the 10–20 configuration. Two electrooculogram (EOG) channels were measured from the right and left outer canthus. Muscle tone was measured using two electromyogram (EMG) electrodes on and under the chin. Lights out occurred between 2200–2300 and lights on between 0600–0700. During sleep, EEG data was monitored by a trained researcher, and the closed-loop stimulation intervention was started when 4 min of continuous visible N2/N3 sleep was observed and allowed to run automatically for the rest of the night unless paused. If the participant showed signs of waking, stimulation was paused and then resumed after the participant returned to N2/N3 sleep. Both verum and control CL-tACS was delivered over F3 and F4 in phase with ongoing SWOs and 180 degrees out of phase with electrodes placed on left and right mastoids. 

The CL-tACS intervention for electrical augmentation of SWOs was developed in MATLAB 2016a (The MathWorks, Inc., Natick, MA, USA) and using EEGLab [20] functions, and is described in detail in [17]. The closed-loop algorithm first detects the presence of SWOs, which consist of slow synchronized positive and negative deflections of EEG that are associated with memory consolidation. The algorithm next attempts to match the stimulation frequency and phase with ongoing slow-wave activity such that the applied sinusoidal waveform matches the temporal dynamics of the endogenous SWOs. For robust SWO detection, a virtual channel is computed by averaging 13 fronto-parieto-central EEG channels (Cz, FC1, FC2, CP1, CP2, Fz, C4, Pz, C3, F3, F4, P3, P4 in the extended 10/20 system) to determine the overall synchronous activity of EEG recorded during sleep. The included channels are stored in a running 5 s buffer. They undergo moving average subtraction with a 1 s window (to mean center the signals at 0 μV), and noisy channels exceeding 500 μV min-to-max amplitude across the 5 s are rejected before the virtual channel is computed. The buffer is updated with each discrete data fetch operation that gets the latest data till the point of data request. By the time the buffer is updated, there is a random transmission delay, which is accounted for to precisely time the brain stimulation intervention. 

The virtual channel data in the buffer is further processed to detect the presence of SWOs so that it can predict when to deliver stimulation to be aligned with ongoing SWOs. The algorithm applies a Fast Fourier Transform (FFT) to these stored data to determine the power spectrum. Stimulation is planned when the ratio of the cumulative power in the SW band (0.5–1.2 Hz) is more than 20% of the total cumulative power from 0.1 to 250 Hz. If this SWO relative power threshold of 0.2 (or 20%) is crossed, the algorithm then filters the data in the SW band with a second-order zero-lag Butterworth filter. Next a sine wave is fit to the filtered virtual channel using the identified dominant frequency in the SW band, and with the amplitude, offset, and phase parameter values optimized. The sine wave is then projected into the future, identifying the temporal targets that would synchronize brain stimulation to the predicted endogenous signal. 

Throughout this process, the dynamic latency associated with data processing is timed using the system clock. Together with distributions of calibrated latencies for data fetch and stimulation commands (mean = 5 ms, SD = 2 ms), which were measured offline, the algorithm determines the correct time point to communicate with the hardware to initiate the simulation. tACS is applied for 5 cycles at the detected SWO frequency. Once stimulation is delivered, the system idles for 3 s to avoid the collection of stimulation artifacts in the data buffer, then resumes the cycle of data collection, detection of SWOs and stimulation planning as per the criteria mentioned above. On control nights, SWOs were detected and stimulation planned in the same way, but 0 mA of current was given. All equipment and staff present on the verum condition were also present for the control condition, the only difference being that no stimulation was applied.

Sleep data were sampled at 500 Hz, and visually assigned a stage (wake, NREM 1, NREM 2, SWS, and rapid eye movement (REM)) per each 30 s epoch, in agreement with the American Academy of Sleep Medicine scoring guidelines [21]. Total sleep time (TST; calculated as the time between sleep onset and sleep offset minus awakenings), minutes of each stage of sleep, and sleep efficiency (calculated as the ratio of time spent asleep out of time in spent bed) were extracted for each participant on each night of in-laboratory sleep. The Karolinska Sleep Diary was completed by participants the following morning to measure subjective sleep quality, sleep onset latency, and TST as well [10].

### 2.4. Analytic Strategy

A repeated measures analysis of variance (RMANOVA) was used to analyze the data using subjective sleep quality as the dependent variable within-subjects, taken at 4 different time points (pre-adaptation night, night 1/adaptation, verum stimulation night, and control stimulation night). The same was done for sleep efficiency at the same time points except the pre-adaptation night, for which the data was not available. Additional RMANOVAs were run using chronological night as the within-subjects dependent variable (pre-adaptation, night 1/adaptation, Night 2, and Night 3 for sleep quality; night 1, Night 2, and Night 3 for sleep efficiency). In these analyses the order of conditions was also accounted for (receiving verum or control stimulation on Night 2 and the opposite condition on Night 3) by using it as a between-subjects independent variable. Sensitivity analyses were performed on sleep quality excluding the 5 participants receiving 0.52 mA on verum nights. No significant differences were found in the main outcome measures between groups receiving high and low CL-tACS (*p* > 0.05). Therefore, data from these participants were included in subsequent analyses. 

A mixed model estimating the effect of the number of stimulation events in predicting subjective sleep quality was performed. The model entailed allowing the slope and intercept to vary for each group on each night. This was done in the R package “Lavaan” as per the methods of [22] to estimate a mixed model within the structural equation modeling framework. Both the significance of the *p*-value and the variance explained using R^2^ were assessed. 

Note that all subjects received the same experimental procedures before both verum and control nights, aside from tDCS during training which was matched by condition to verum vs. control used that evening. Therefore, there were no differences in behavioral treatments that may have influenced the differences found between the verum and control nights. Both control and verum stimulation were administered by a small team of highly trained staff, and sleep scoring was performed by a single rater.

## 3. Results

**Figure 1:**
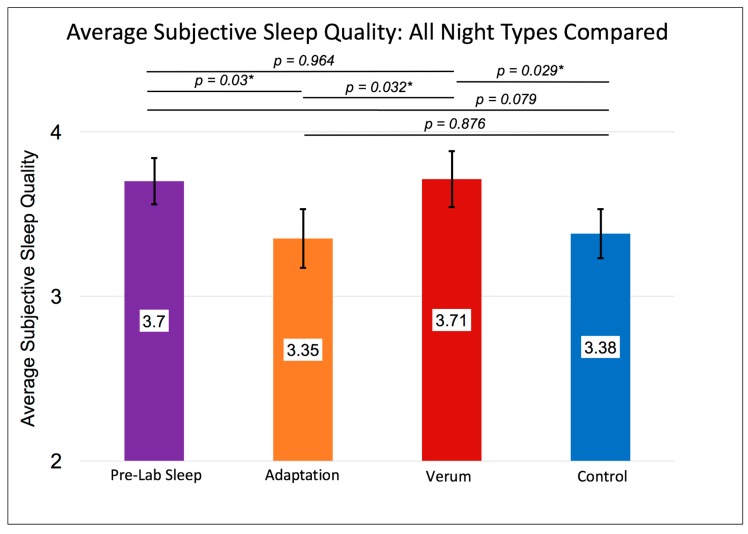
Shows the within-subjects contrast of sleep at home before coming to the laboratory (purple), adaptation night 1 (orange) and verum (red bar) vs. control (blue bar) stimulation collapsed across nights 2 and 3 on subjective sleep quality, *n* = 19. Error bars represent +/- 1 standard error of the mean. Results of individual, uncorrected *t*-tests are shown at the top. An asterisk “*” indicates a significant difference (*p* < 0.05). The omnibus multivariate F was significant at *p* = 0.021. The within-subjects’ F was significant at *p* = 0.04. Subjective ratings of sleep quality on the Karolinska Sleep Diary indicate comparable sleep quality on the verum night as during pre-laboratory (home) sleep and significantly greater sleep quality on verum nights compared to adaptation or control nights.

Prior to analyses, each dependent variable was investigated for normality. No outliers were detected. Due to missing data for some dependent variables, sample size varied by model. Subjective sleep quality results were analyzed using 19 of 21 participants for whom the sleep quality data was available for each of the four nights (see Figure 1). Calculating sleep efficiency requires computing the ratio of time spent asleep (as determined by sleep staging from EEG) out of total time in bed. Seven participants had excessive noise in sleep EEG for one or more nights for accurate sleep staging, so sleep efficiency results were limited to 14 participants. 

**Figure 2:**
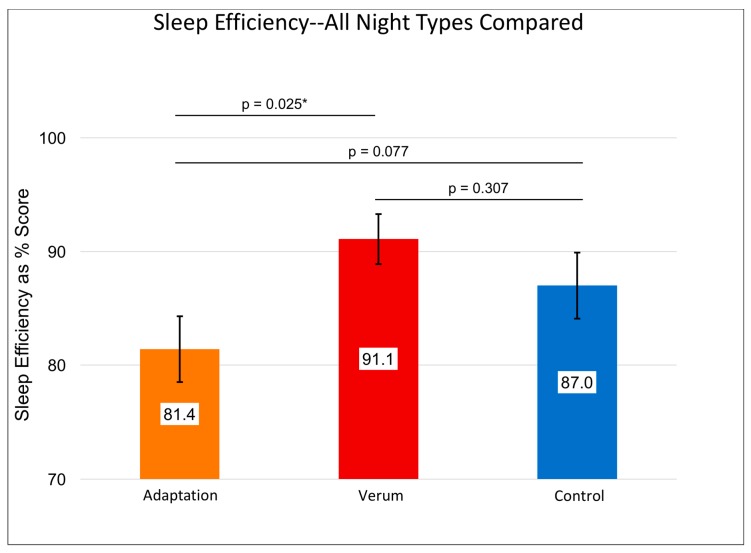
Shows the repeated measures effects adaptation night 1 (orange) and verum (red bar) vs. control (blue bar) stimulation collapsed across nights 2 and 3 on sleep efficiency, *n* = 14. Results of individual, uncorrected *t*-tests are shown at the top. An asterisk “*” indicates a significant difference (*p* < 0.05). The omnibus within-subjects’ F was significant at *p* = 0.038. Error bars represent +/- 1 standard error of the mean. Sleep efficiency variables only exist for nights of in-laboratory sleep, so no “Pre-Lab Sleep” measure could be obtained. Sleep efficiency for verum night was significantly greater than that for the adaptation night.

Without accounting for the counterbalancing of stimulation condition order, a RMANOVA investigating differences in subjective sleep quality across nights showed a significant multivariate effect (F (3,15) = 4.273, *p* = 0.021, Figure 1) and a significant within-subjects effect (F (3,15) = 2.955, *p* = 0.040). These results were driven by higher sleep quality rated after nights of verum stimulation compared to control nights (M_Verum_ (SE) = 3.71 (0.17), M_Control_ (SE) = 3.38 (0.15), *p* = 0.029; Paired Samples Cohen’s *d* = 0.47) and compared to adaptation nights (M_Verum_ (SE) = 3.71 (0.17), M_Adaptation_ (SE) = 3.35 (0.18), *p* = 0.032, Paired Samples Cohen’s *d* = 0.48). See Figure 1 for details. Sleep efficiency followed a similar pattern (Figure 2) whereby the Greenhouse-Geisser corrected, within-subjects F was significant (F (1.803,13.53) = 3.735, *p* = 0.043), with higher sleep efficiency on verum nights compared to adaptation nights (M_Verum_ (SE) = 0.911 (0.022), M_Adaptation_ (SE) = 0.814 (0.029), *p* = 0.025, Paired Samples Cohen’s *d* = 0.87). 

We used *t*-tests to explore contrasts within the omnibus effect of time represented in Figure 1 and Figure 2. These pairwise comparisons were planned, a priori contrasts and therefore were not alpha-corrected. In the RMANOVA results accounting for stimulation order, the differences in sleep quality between the verum and control nights for the V/C group would remain significant even if using a Sidak-corrected alpha level of *p* < 0.0051 for 10 unplanned comparisons (mean difference on sleep quality in V/C group verum vs. control night = 3.82, *p* = 0.005). 

**Figure 3:**
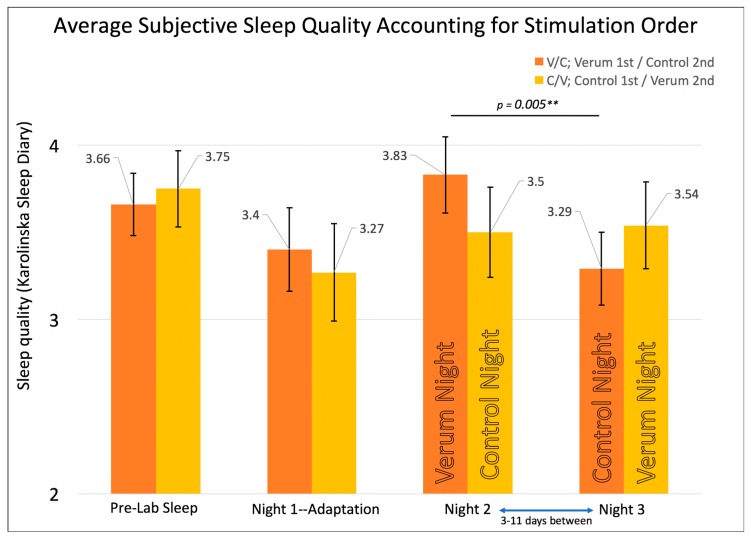
Shows the repeated measures effects of sleep quality while accounting for counterbalancing of stimulation condition order. The omnibus multivariate F was significant at *p* = 0.042. Error bars represent +/- 1 standard error of the mean. Significant differences were found between the verum and control nights only for the group receiving verum stimulation on Night 2 (namely, the Verum/Control V/C group), with a double asterisk “**” indicating *p* = 0.005 for an individual, uncorrected *t*-test.

In order to examine the effects of stimulation condition order, participants were divided into groups receiving verum first on Night 2 (V/C) or control first on Night 2 (C/V). The RMANOVA on subjective sleep quality (*n* = 19; V/C = 11; C/V = 8) showed a significant multivariate F (F (3,15) = 3.494, *p* = 0.042), prompting contrasts of groups at each time point and the time points within each group. There was a significant difference within the V/C group between their verum night and control night on subjective sleep quality (Figure 3) with higher sleep quality after verum stimulation (M_Night2forV/C—(Verum)_ (SE) = 3.83 (0.22), M_Night3forV/C—(Control)_ (SE) = 3.29 (0.21), *p* = 0.005, Paired Samples Cohen’s *d* = 0.82). By contrast, there were no significant differences on subjective sleep quality within the C/V group on their verum nights compared to their control nights (M_Night2forC/V—(Control)_ (SE) = 3.54 (0.25), M_Night3forC/V—(Verum)_ (SE) = 3.50 (0.26), *p* = 0.86).

**Figure 4:**
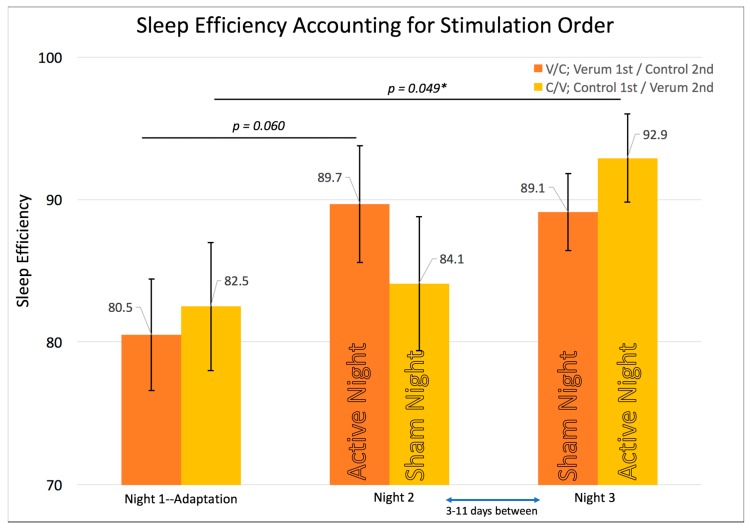
Shows the repeated measures effects of sleep efficiency while accounting for the counterbalancing of stimulation condition order. Error bars represent +/- 1 standard error of the mean. Results of individual, uncorrected *t*-tests are shown at the top. An asterisk “*” indicates a significant difference (*p* < 0.05). The omnibus multivariate F was significant at *p* = 0.044. The within-subjects’ F was significant at *p* = 0.049. Sleep efficiency shows the highest scores on each group’s verum night with a trend for the V/C group’s verum night vs. adaptation night and a significant effect for the Control/Verum C/V group’s verum night vs. adaptation night.

Initial attempts at elucidating the effect of our intervention led to modeling subjective sleep quality from sleep efficiency. This revealed that that sleep efficiency was especially predictive of sleep quality in the V/C group for the night of verum stimulation, which occurred immediately after the adaptation night. The same RMANOVA model was tested using sleep efficiency as the dependent variable (*n* = 14; V/C = 8; C/V = 6) at each time point instead of sleep quality (see Figure 4). Results showed a significant multivariate test (F (2,11) = 4.198, *p* = 0.044) and a significant within-subjects test (F (2,11) = 3.418, *p* = 0.049), prompting contrast *t*-tests therein. The only significant contrast existed for the C/V group showing the highest sleep efficiency on Night 3, when that group received verum stimulation compared to the adaptation night/night 1 (M_Night3forC/V-(Verum)_ (SE) = 0.93 (0.03), M_Night1forC/V-(Adaptation)_ (SE) = 0.83 (0.05), *p* = 0.049, Paired Samples Cohen’s *d* = 0.96). Also noteworthy is that sleep efficiency peaked for both groups on their night of verum stimulation rather than at any other night in the laboratory (Figure 4). To ensure that the effect on sleep efficiency was due to stimulation rather than experimenter error (for example, accidentally getting participants to bed sooner on verum stimulation nights), a series of RMANOVAs were conducted on sleep variables such as self-reported and objectively measured TST for each time point, as well as the precise time at which lights off and on occurred. Across all four variables that might affect sleep efficiency, there were no significant differences. See Table 1 for total sleep time and individual sleep stage across conditions and nights.

The mixed model used the maximum likelihood estimator, thereby retaining all available data points for each night. The results of the mixed model confirmed that the effect of the number of stimulation events on sleep quality was positive and significant for the V/C group on Night 2, the night of their verum stimulation (B (SE) = 0.01 (0.004); *p* = 0.019, R^2^ or variance explained in sleep quality = 0.31). There was no positive or significant effect for the C/V group on Night 2 when control stimulation was delivered (i.e., the number of stimulation events as estimated using our software, but no current was delivered; B (SE) = −0.004 (0.004); *p* = 0.27, R^2^ or variance explained in sleep quality = 0.12). This suggests that it is the stimulation rather than the number of endogenous SWOs that is directly involved in this effect. On Night 3, the night without a prior night of re-adaptation to sleep in the laboratory, there was no effect of stimulation events for either the control night for the V/C group (B (SE) = 0.000 (0.002); *p* = 0.82, R^2^ or variance explained in sleep quality = 0.004) or the verum night for the C/V group (B (SE) = −0.001 (0.013); *p* = 0.92, R^2^ or variance explained in sleep quality = 0.001). This confirms the RMANOVA results for sleep efficiency and sleep quality reported above.

## 4. Discussion

The present results show that subjective sleep quality and sleep efficiency showed significant improvements associated with a closed-loop tACS intervention designed to match the phase and frequency of endogenous SWOs in NREM sleep. The V/C group showed its highest sleep efficiency and highest rated sleep quality for the night of verum stimulation (Night 2) compared to control (Night 3). Sleep quality and efficiency were larger for the V/C group on Night 2 compared with the C/V group on Night 3, suggesting that a prior night of poor sleep may promote these effects.

Continuous positive airway pressure (CPAP) machines have also been found to improve sleep efficiency and subjective sleep quality in sleep deficient patients [7]. Specifically, less N1 sleep, more SWS, and more REM were associated with greater sleep quality. In another study, hypoglossal nerve electrical stimulation was used to treat sleep apnea [23]. This resulted in a reduced N1 sleep time, increased REM sleep time, and improved subjective sleep quality. These observations suggest our intervention may result in similar sleep quality improvements for sleep-lacking healthy participants as other interventions do for sleep-disordered patients [7,8]. Perhaps it is the duration of time in different sleep stages, and not the TST per se, that may be influenced by stimulation, thereby having an effect on the sleep efficiency and sleep quality. However, the open-loop offset oscillatory-tDCS used by [15] did not result in changes of time spent in N1, N2, or SWS over the course of an afternoon nap, and did not significantly modulate sleep efficiency. Our study, on the other hand, employed closed-loop tACS during a full night’s sleep. Other methods of stimulation designed to enhance memory consolidation, such as closed-loop auditory stimulation [24], have not resulted in changes in sleep quality. This suggests that the neural basis of changes in memory consolidation and sleep quality may be different, given that they can be affected separately using different forms of stimulation.

### Limitations

There are several limitations in the current design, the most obvious being the low sample size available for this pilot study analysis. Lower sample sizes can lead to more imprecision of mean scores and therefore larger standard errors, which may explain the lack of significant findings between V/C and C/V groups within each night. This is somewhat supported by the significant results found after pooling the experimental nights together, comparing verum and control conditions regardless of counterbalancing of stimulation condition order, thereby increasing the available sample size (*n* = 19) and reducing standard errors of the mean. Power analyses indicate that *n* = 64 would be needed per group to detect the current between-group order effects with 80% power (Independent Samples Cohen’s *d* = 0.44; Figure 3). 

This study was conducted as part of a larger trial to use transcranial electrical stimulation to enhance memory consolidation [17], which entailed the use of tDCS during waking/daytime afternoon training sessions combined with nighttime CL-tACS to maximize effects on performance. It may be possible that the daytime tDCS also increased sleep quality [13]. However, evidence from the tDCS literature suggests that prefrontal tDCS may have the opposite effect on sleep quality from what we see in our results. [25] found that anodal stimulation to the dorsolateral prefrontal cortex (DLPFC) resulted in reduced sleep efficiency for their sample. Therefore, the presence of CL-tACS related improvements in sleep efficiency after the application of frontal cortex tDCS exposure in the present study is interesting given the potential negative effects of tDCS applied the afternoon before.

Finally, the greater number of technical difficulties in the group receiving verum stimulation on their third night (namely, the C/V group) led to reduced number of subjects in this group, and the reduction in statistical power may be one reason why results in this group were inconclusive. This may also be due to lack of an adaptation night to in-laboratory sleep after 3–11 days of sleep at home. Thismay cause a “first night effect” [26,27,28] on Night 3 for the C/V group in which sleep quality might be harmed by lack of adaptation. Alternatively, for the V/C group, having an adaptation night prior to being treated with stimulation the night thereafter may result in creating sleep deficits on the adaptation night for which the intervention may be corrective on the subsequent night. 

## 5. Conclusions

This study shows promising results that CL-tACS designed to match the phase and frequency of endogenous SWOs during sleep may improve sleep efficiency that night, as well as self-reports of sleep quality the next day. This effect was found to be larger following a night of poor sleep. These results provide further evidence that SWOs are related to sleep efficiency and sleep quality, in that manipulating SWOs was correlated with positive changes in these measures. Additional work with larger sample sizes will be necessary to pinpoint the exact changes in sleep stages and the EEG effects of stimulation that are related to these self-reports of improvements in sleep quality. If confirmed, this technique offers the possibility of a novel, non-pharmacological method for improving sleep quality and efficiency, which may eventually provide a benefit to reduce sleep deficits in healthy people, and a new form of treatment for illnesses that involve sleep deficits.

## Figures and Tables

**Table 1:** Mean Values on Sleep Quality, Sleep Efficiency, N1, N2, Slow-Wave and, REM Sleep Time for Each Group Per Each Night of In-Laboratory Sleep.

	V/C on Night 1 Mean (SD)-Adaptation Night	C/V on Night 1 Mean (SD)-Adaptation Night	V/C on Night 2 Mean (SD)-Verum Night	C/V on Night 2 Mean (SD)-Control Night	V/C on Night 3 Mean (SD)-Control Night	C/V on Night 3 Mean (SD)-Verum Night
Subjective Sleep Quality	3.4 (0.53)	3.27 (1.05)	3.83 (0.53)	3.5 (0.95)	3.29 (0.38)	3.53 (0.98)
Sleep Efficiency (%)	81 (12)	82 (9)	89 (11)	84 (12)	89 (10)	92 (2)
N1 Sleep Time (min)	23.94 (11.50)	19.33 (6.94)	16.31 (8.24)	19.42 (8.77)	26.31 (14.44)	18.58 (9.44)
N2 Sleep Time (min)	219.81 (73.40)	279.00 (60.95)	241.13 (72.56)	242.17 (77.78)	254.13 (74.50)	274.75 (32.63)
SWS Sleep Time (min)	72.81 (38.57)	76.75 (13.22)	70.63 (30.80)	90.17 (35.45)	77.06 (29.27)	98.50 (8.29)
REM Sleep Time (min)	53.31 (19.71)	55.50 (20.39)	64.31 (34.48)	47.33 (23.46)	49.63 (22.66)	59.75 (23.60)
Total Sleep Time (min)	369.88 (89.65)	368.08 (59.56)	392.38 (92.12)	399.08 (108.94)	407.13 (81.78)	451.58 (45.10)

Values of sleep time for the stages are in minutes, sleep efficiency is scored as a percentage, and subjective sleep quality ranges from a minimum score of 1 to a maximum score of 5, with 5 being the best. SWS; slow-wave sleep; REM: rapid eye movement; V/C: Verum/Control; C/V: Control/ Verum.
